# ExPert ConsEnsus on the management of Advanced clear-cell RenaL celL carcinoma: INDIAn Perspective *(PEARL-INDIA)*

**DOI:** 10.1186/s12885-023-11237-y

**Published:** 2023-08-09

**Authors:** Tarini Prasad Sahoo, Chirag Desai, Shyam Agarwal, Amit Rauthan, Boman Dhabhar, Ghanshyam Biswas, Sandeep Batra, Rajat Saha, Arun Philip, Vijay Agarwal, Palanki Satya Dattatreya, PN Mohapatra, Chetan Deshmukh, Sagar Bhagat, Saiprasad Patil, Hanmant Barkate

**Affiliations:** 1 Medical Oncology, Silver Line Hospital, Bhopal, Madhya Pradesh India; 2 Medical Oncology & Director Hemato-Oncology Clinic Vedanta, Ahmedabad, Ahmedabad India; 3 Medical Oncology, Sir Gangaram Hospital, Delhi, India; 4 Medical Oncology, Manipal Hospital, Bangalore, India; 5 Medical & Hemat-Oncology, BND Onco Center, Mumbai, India; 6 Sparsh Hospitals & Critical Care, Odisha Bhubaneswar, India; 7 Medical Oncology, Max Superspeciality Hospital, Saket, New Delhi, India; 8 Medical Oncology Amrita Institute of Medical Sciences, Cochin, India; 9 Medical Oncology Aster, CMI Hospital, Bangalore, India; 10 Medical Oncology Services Renova Hospitals, Hyderabad, India; 11 Medical Oncology, Apollo Hospital, Kolkata, India; 12 Medical Oncology, Deenanath Mangeshkar Hospital, Pune, India; 13 DGM, Global Medical Affairs, Glenmark Pharmaceutical Limited, B D Sawant Marg, Chakala, Andheri East, Maharashtra 400099 Mumbai, India; 14 GM, Global Medical Affairs, Glenmark Pharmaceutical Limited, Mumbai, India; 15 Medical Affairs, Glenmark Pharmaceutical Limited, Mumbai, India

**Keywords:** Renal cell carcinoma, Tyrosine kinase inhibitors, Immunotherapy, Consensus, Cabozantinib

## Abstract

In advanced Renal Cell Carcinoma (aRCC), systemic therapy is the mainstay of treatment, with no or little role for surgery in these patients. Tyrosine kinase inhibitors (TKIs) and immune-oncological (IOs) therapies, either alone or in combination, are recommended in these patients depending on patient and tumour factors. The sequencing of therapies is critical in RCC because the choice of subsequent line therapy is heavily dependent on the response and duration of the previous treatment. There are additional barriers to RCC treatment in India. Immunotherapy is the cornerstone of treatment in ccRCC, but it is prohibitively expensive and not always reimbursed, effectively putting it out of reach for the vast majority of eligible patients in India. Furthermore, in advanced RCC (particularly the clear cell variety), Indian oncologists consider the disease burden of the patients, which is particularly dependent on the quantum of the disease load, clinical symptoms, and performance status of the patient, before deciding on treatment. There are no India-specific guidelines for clear cell RCC (ccRCC) treatment or the positioning and sequencing of molecules in the management of advanced ccRCC that take these country-specific issues into account. The current consensus article provides expert recommendations and treatment algorithms based on existing clinical evidence, which will be useful to specialists managing advanced ccRCC.

**Supplementary Information:**

The online version contains supplementary material available at 10.1186/s12885-023-11237-y.

## Introduction

Renal cell carcinoma (RCC) constitutes almost 3% of all cancers. RCC is the most frequent solid tumor in kidney which accounts for almost 90% of all kidney malignancies with clear-cell renal cell carcinoma (ccRCC) being the most common type [1, 2]. The yearly incidence of kidney cancer in India is 16,861 with a 5-year prevalence of 2.84/ 100,000 population [3]. However, mortality rates in developing countries like India are higher as compared to that of the developed countries [4]. Nearly one-third of the RCC patients present with advanced disease at diagnosis and almost one-third of the localized RCC patients treated with curative intent ultimately progress to the advanced stage [5, 6]. Moreover, advanced RCC has poor prognosis, with a 5-year survival rate as low as 8% when compared with an overall rate of 74% for all RCCs [7, 8]. Sarcomatoid histology constitutes almost 15% of all RCC cases which can be seen either as a separate entity or as a sarcomatoid differentiation together with other histologic subtypes and portends an especially poor prognosis and have shown responses to immunotherapy regimens [9, 10].

Systemic treatment forms the mainstay of treatment in advanced RCC with no or minimal role of surgery in these patients [6]. The topography of systemic therapies has evolved rapidly over the past few years [11]. ccRCC is a highly vascularized tumor characterized by an increase in the level of angiogenic factors, including vascular endothelial growth factor (VEGF). Hence, anti-VEGF therapies have a noteworthy role in the treatment of advanced ccRCC and have replaced the earlier standard of therapies such as interferon (IFN)-α and interleukin-2 that were in use 2 decades ago [9]. Moreover, ccRCC is also a highly immunogenic cancer distinguished by an affluence of immune cells leading to an increased purpose for the use of immune-oncological (IO) therapies. As a consequence of this, treatment options for advanced ccRCC include agents that target angiogenesis pathway or IO pathways or both. These include VEGF receptor (VEGFR) targeting tyrosine kinase inhibitors (TKIs) such as cabozantinib, lenvatinib, axitinib, pazopanib and sunitinib, mammalian target of rapamycin (mTOR) inhibitors namely everolimus and immune check point inhibitors (ICIs) acting as anti-PD-1 such as pembrolizumab, avelumab and nivolumab, or anti-CTLA-4 ICI namely ipilimumab [12, 13].

TKIs and IOs as single agent or in combination are recommended for advanced RCC depending upon various factors (patient and tumor related) [14]. Choice of treatment is often governed by prognostic factors such as performance status, laboratory parameters, prior history of nephrectomy, etc. [15]. The recent guidelines for advanced ccRCC treatment [16, 17] mention about preferred and alternative first line and second line systemic treatment options for ccRCC but optimal sequencing of therapies still remains a dilemma. Besides, majority of the immunotherapies and TKIs for RCC are approved very recently with lack of direct comparison among themselves. To add to this, there is evidence to suggest that as high as 50% of patients with metastatic RCC receive a second-line therapy, thus clinicians need to be familiar with the clinical and molecular aspects of each therapy used in the various lines of treatment [18, 19]. Hence, sequencing of therapies is very important in RCC as the subsequent line therapy choice is hugely depends upon the response and duration of the same to the previous treatment [20].

Additionally, in India there are further barriers to RCC treatment. Immunotherapy forms the cornerstone of treatment in ccRCC, but immunotherapy is available at a considerably high price [21, 22] and is not always reimbursed, practically making it out of reach of the majority of the eligible patients. Although the guidelines recommend about upfront use of immunotherapy and TKI as a combination therapy, use of single agent TKI is still practiced in the Indian setting [22]. Moreover, in advanced RCC (specially the clear cell variety), apart from the risk stratification criteria, experts were also of the opinion that Indian oncologists also take into account the disease burden of the patients, which particularly depends upon the quantum of the disease load, clinical symptoms and performance status of the patient before deciding the treatment. Hence, it will also be important to gauge the choice of treatment based on disease burden. In spite of the regulatory approval of the different TKIs and immunotherapies in India, there are no India specific guidelines for ccRCC treatment and the positioning as well as sequencing of molecules in the management of advanced ccRCC, which takes into account these country specific issues [19]. In the above context, an exercise was done to arrive at a consensus regarding sequencing of systemic therapies in the management of advanced ccRCC using the modified Delphi method. The current consensus article provides expert recommendations and treatment algorithms based on the existing clinical evidence which will be of aid to specialists involved in the management of advanced ccRCC.

## Methodology

This consensus document was developed using a modified Delphi method by a geographically diverse panel of subject experts from April 2022 to September 2022. The class of recommendation and level of evidence grading used in this manuscript are based on the grading system used by Knuuti et al. which was modified for suitability in the current study. The same has been depicted in Table 1 below [23]. The class of recommendations and level of evidence are two independent evaluations, thereby allowing a strong recommendation even in the absence of the highest quality evidence.

**Table 1:** Class of recommendation and level of evidence

Class of Recommendation		Consensus Response
I	Evidence and/or general agreement that a given treatment or procedure is beneficial, useful, effective	Consensus (It is recommended or is indicated)
II	Conflicting evidence and/or a divergence of opinion about the usefulness/efficacy of the given treatment or procedure	Near consensus (May be considered)
III	Evidence or general agreement that the given treatment or procedure is not useful/effective, and in some cases may be harmful	No consensus (It is not recommended)
Level of evidence		
A	Data derived from multiple randomized clinical trials or meta-analysis	
B	Data derived from a single randomized clinical trial or large non-randomized studies	
C	Consensus of opinion of the experts and/or small studies, retrospective studies, registries	

**Fig. 1:**
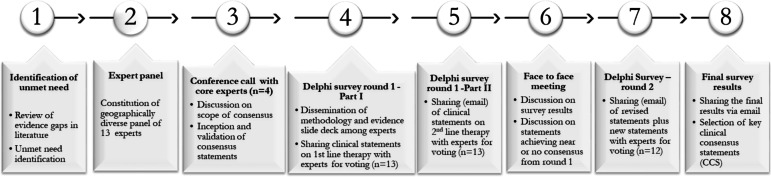
Consensus process using modified Delphi method

Figure 1 provides a brief description of the consensus process used to create the clinical consensus statement (CCS) used in the current manuscript.

Panel of 13 medical oncologists was selected based on clinical experience, academic achievements and engagement in clinical research in the area of RCC which included 4 experts that were a part of the core panel for preparing and reviewing the clinical statements. An electronic search of PubMed and Embase database was conducted in order to develop the clinical statements for the current consensus. A rigorous literature search was carried out to identify the relevant articles written in English and published over the last 15 years between 1 January 2007 to 1 August 2022, using keywords renal cell carcinoma, sequencing, tyrosine kinase inhibitors, immunotherapy, disease burden, sunitinib, pazopanib, axitinib, cabozantinib, lenvatinib, pembrolizumab, avelumab, nivolumab, ipilimumab, everolimus and combination therapy. Final results of the literature search were disseminated among the panel members in the electronic full-text version. Experts were asked to review the articles identified during the literature search to identify the evidence gaps and unmet need for the systemic treatment of aRCC to assist in the development of clinical statements.

Panel members completed two Delphi surveys (89 clinical statements in round one and 21 clinical statements in round two via email, using a 9-point Likert scale). First round of the Delphi survey consisting of 89 statements was disseminated in two parts, i.e., part I for first line therapies and part II for second line therapies in aRCC. This was followed by a face-to-face meeting with the experts, during which results from the first round were presented and clinical statements where ‘no consensus ‘or ‘near consensus’ were achieved were discussed to determine whether they should be refined and added to the Delphi survey or omitted completely. Criteria used for this includes – Clinical practice relevance of the statement, Look back at evidences for and against the statement, Personal opinion of the experts. Later on, the redefined clinical statements were shared with core experts for evaluation and approval and later were shared with all expert as a part of Delphi round 2.

Finally, algorithms for the sequencing of treatment for advanced ccRCC were prepared based on the consensus statement responses and the available clinical evidence. Post the meeting, 21 clinical statements were re-framed or formulated to be taken for second Delphi round. 13 experts participated in the first round of Delphi survey while 12 experts participated in second round of Delphi survey. Class of recommendation was based on a 9-point Likert scale [24] and was as follows:

- Consensus: Statements achieving a mean score of 7.00 or higher and having no more than one outlier
- Near consensus: Statements achieving a mean score of 6.50 or higher and having no more than two outliers
- No consensus: Statements that did not meet the criteria of consensus or near consensus

Statistical analysis: Responses from both rounds of the survey were collected and analyzed by the chair and staff liaison. Descriptive statistics were calculated for each statement to include the mean score and outliers. Outlier was defined as any rating ≥ 2 Likert points from the mean in either direction. Statements were further grouped into the corresponding class of recommendation.

Ethics committee approval was not required for this methodology because this was a modified Delphi-based consensus document with no human/subject involvement (active/passive) or use of human tissue samples. Furthermore, all of the data utilised to generate the consensus guideline were publicly available in the public domain and did not include any mention of new drugs (as defined by CDSCO). The Indian national ethical guideline, ICMR, [25] additionally stated that ethics committee permission is required in any biomedical, social, and behavioural health research involving human participants and biological material. As a result, ethics committee approval was not required for the methodology we utilised, which involved voting among recognised experts in the field. However, all experts who participated in this consensus process were aware about the objectives of the study, and the participants were also aware that this consensus document would be utilized for publication purposes. Participant consent was taken prior to dissemination of the survey. Participants provided independent responses on the Delphi survey based on their previous clinical experience. The final responses of all expert participants were analyzed to calculate mean and outliers for each consensus statement. All methods were carried out with adhering guideline and regulation.

## Results

**Fig. 2:**
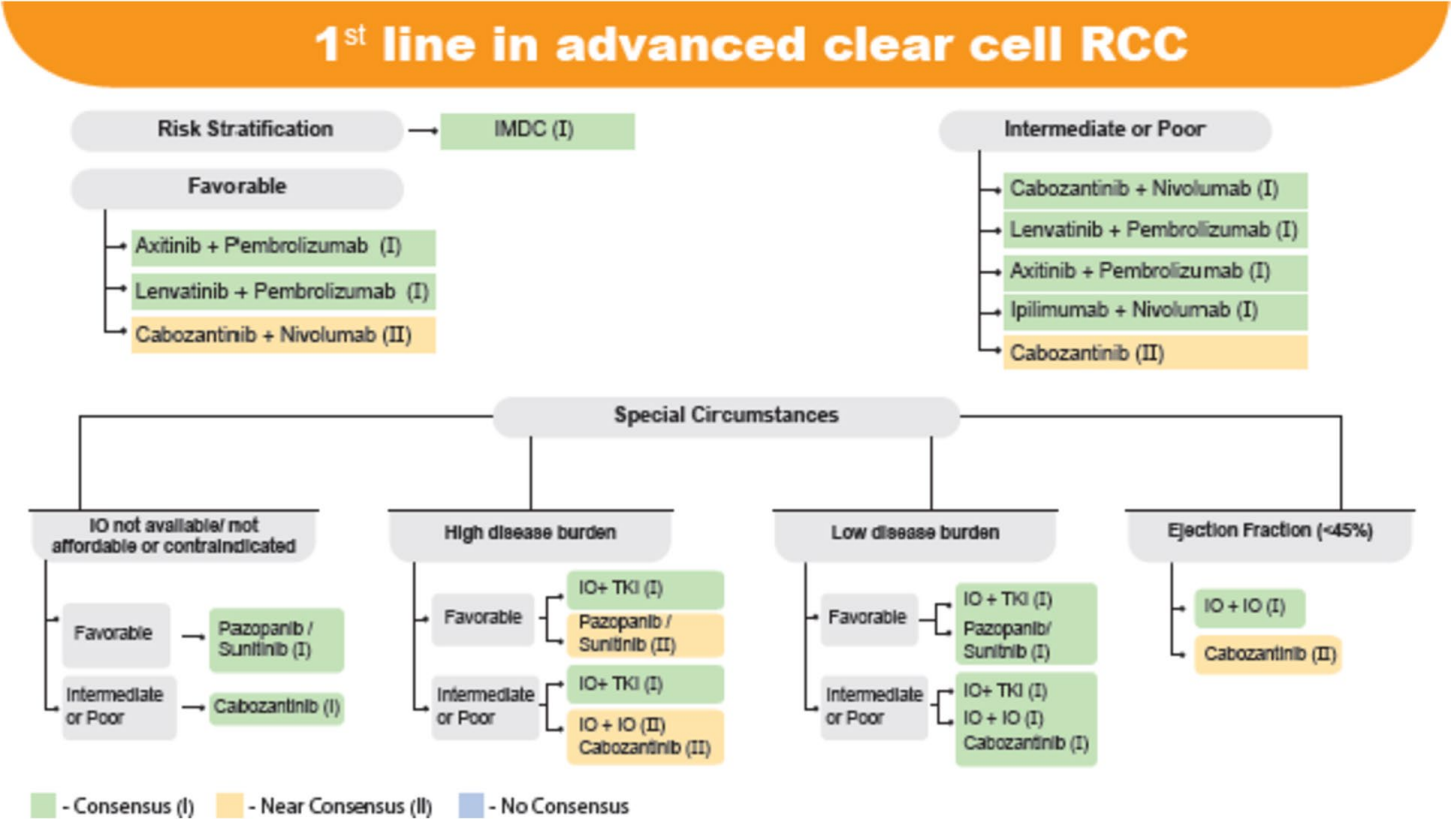
First line treatment in advanced clear cell RCC

**Fig. 3:**
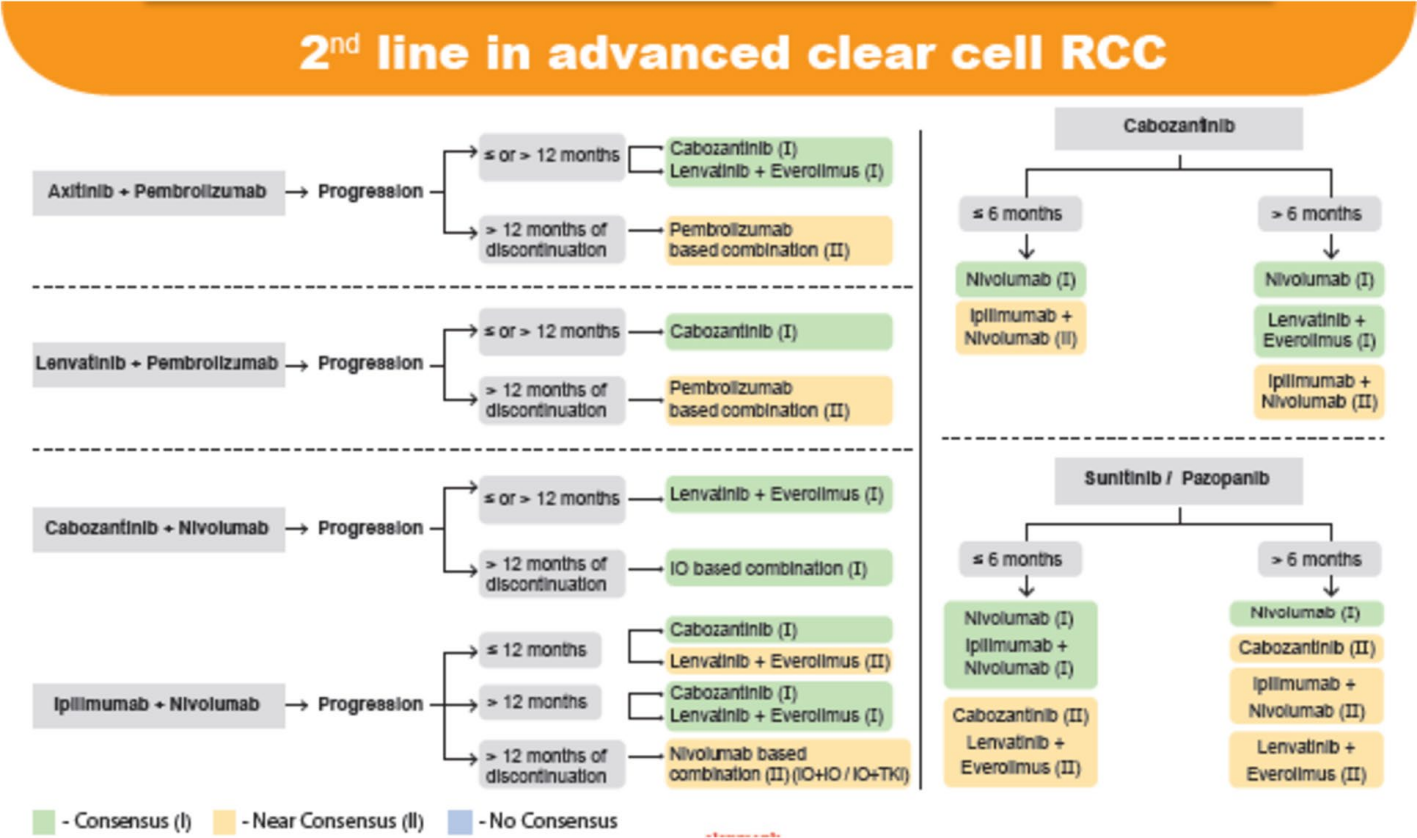
Second line treatment in advanced clear cell RCC

During the consensus process, the expert panel engaged in a thorough discussion and evaluated relevant evidences. Experts provided recommendations on 89 clinical statements that were categorized into 3 groups: Risk stratification (2 statements), First line therapy (36 statements), and Second line therapy (51 statements). A summary of key recommendations based on expert responses and level of evidence is represented in Figs. 2 and 3. Furthermore, of the 89 clinical statements, 22 statements laid emphasis on RCC treatment selection in special circumstances including non-availability or non-affordability or contraindication of immune (IO) therapy, high and low disease burden, cardiovascular comorbidity), which has been depicted in Table 2. The class of recommendation (COR) and level of evidence (LOE) for each statement has been mentioned in the supplementary appendix.

**Table 2:** First line systemic therapy for accRCC in special circumstances

	**Clinical statement**	**Mean score**	**Outliers**	**Consensus result**	**COR and LOE**
**I**	**Preferred clinical practice therapy when immune therapy is not available/not affordable or is contraindicated**				
	***Favourable risk category***				
1	Pazopanib/ Sunitinib is the preferred therapy	8.31	1	Consensus	I-A
	***Intermediate/poor risk category***				
1	Pazopanib/ Sunitinib is the preferred therapy	5.77	4	No consensus	III-A
2	Cabozantinib is the preferred therapy	7.31	1	Consensus	I-A
**II**	**Preferred class of therapy/therapy in clinical practice in high disease burden: Symptomatic and/or rapidly progressive disease requiring rapid control** (In disease burden categorization stated above, type and nature of symptoms to be judged clinically along with risk stratification while managing the patient)				
	***Favourable risk category***				
1	TKI (Pazopanib/sunitinib) is the preferred therapy	7.08	2	Near consensus	II- C
2	IO + TKI is the preferred class of therapy	8	0	Consensus	I- C
3	IO + IO is the preferred class of therapy	5.31	5	No consensus	III- C
	***Intermediate/poor risk category***				
1	TKI (Pazopanib/sunitinib) is the preferred therapy	5.38	8	No consensus	III- C
2	TKI (Cabozantinib) is the preferred therapy	7.46	2	Near consensus	II- C
3	IO + TKI is the preferred therapy	8.46	0	Consensus	I- C
4	IO + IO is the preferred class of therapy	6.85	1	Near consensus	II- C
**III**	**Preferred therapy/class of therapy in clinical practice in low disease burden**				
	***Favourable risk category***				
1	TKI (Pazopanib/sunitinib) is the preferred therapy	8.31	0	Consensus	I- C
2	IO + TKI is the preferred class of therapy	7.77	1	Consensus	I- C
3	IO + IO is the preferred class of therapy	5.08	5	No consensus	III-C
	***Intermediate/poor risk category***				
1	TKI (Pazopanib/sunitinib) is the preferred therapy	5.54	6	No consensus	III-C
2	TKI (Cabozantinib) is the preferred therapy	7.38	1	Consensus	I-C
3	IO + TKI is the preferred class of therapy	8	1	Consensus	I-C
4	IO + IO is the preferred class of therapy	7.08	1	Consensus	I-C
**IV**	**Preferred clinical practice therapy in cardiovascular comorbidity (ejection fraction < 45%) irrespective of risk stratification**				
1	IO + TKI is preferred	6.31	1	No consensus	III-C
2	IO + IO is preferred	7.15	1	Consensus	I-C
3	Single agent TKI (Cabozantinib) is preferred	6.62	1	Near consensus	II-C
4	Single agent TKI (Pazopanib) is preferred	6.23	2	No consensus	III-C
5	Single agent TKI (Sunitinib) is preferred	4.54	1	No consensus	III-C

## Discussion

The treatment for advanced clear cell RCC (ccRCC) has evolved in the recent decades due to the availability of several new molecules especially immunotherapy. This expert consensus focuses on the treatment selection and sequencing strategies for advanced ccRCC based on the available evidence disease burden, affordability, comorbidities and symptoms of the patients in Indian setting.

### Risk stratification criteria

The risk stratification models for mRCC were developed primarily with an intent to predict individual patient prognosis [26]. The International Metastatic renal cell carcinoma Database Consortium (IMDC) and Memorial Sloan- Kettering Cancer Center (MSKCC) scoring criteria are two most widely accepted and internationally validated models for risk stratification in mRCC. Both of the above models are used as a prognostic index to stratify patients into three subgroups: Favourable/ Good, intermediate and poor-risk groups [26, 27].

According to the IMDC criteria [28], prognostic factors such as Karnofsky score-based performance status and laboratory parameters such as Serum calcium, Blood Haemoglobin, platelet and Neutrophil are parameters considered in risk stratification; MSKCC considers LDH in place of Platelet and Neutrophil. Either of the criteria is applied on a global scale. The 1^st^ step in initiating systemic treatment in patients with aRCC is risk assessment. According to the IMDC criteria, scores are assigned to the parameters, and patients are classified as favourable (scoring -0), intermediate (score 1–2), or poor (score ≥ 3) prognostically based on the existence of elevated or lowered lab parameters and an 80% Karnofsky score. Risk stratification models has demonstrated a predictive capability in the context of these treatments including immune checkpoint inhibition. The treatment is then determined accordingly.

Systemic inflammatory markers, which are included in the IMDC risk model but not the MSKCC risk model, are useful predictors, particularly for the poor prognosis category [29]. It is important to appropriately classify poor risk patients as patients with poor risk have a short survival expectancy and unlike the case with low risk patients, cytoreductive nephrectomy may not be the primary treatment in poor risk patients [30].

### Consensus recommendation

In the current consensus, the expert panel highlighted important points in support of IMDC. The inclusion of easily assessable parameters in IMDC such as blood counts, clinical features and corrected calcium levels, apart from the evidence in the recent publications with TKIs, weighs it over MSKCC.

### Active surveillance

An active surveillance-based approach at the start of treatment has been explored prospectively in the management of breast cancer [31]. However, there is relatively limited prospective evidence for active surveillance in RCC patients [32]. Among patients with mRCC, there is a subset with slow-growing metastases for whom systemic therapy can be safely delayed and active surveillance offered sparing treatment related toxicity without affecting the survival while preserving the quality of life (QoL). Rarely do these therapies lead to complete responses that allow for permanent treatment discontinuation, which suggests that most patients receiving systemic therapies will be treated indefinitely, sequencing from one therapy to the next. These patients have not been well defined because contemporary mRCC trials do not include an arm without treatment. Recognizing that some of these patients may not require systemic therapies for months or years can be an important and probably makes AS a discussion point with the right patients. Currently, the NCCN guidelines list AS as a level 2A recommendation in selected patients, but with limited supportive evidence.

In a recent observational study by Harrison et al. the median overall survival was not reached (95% CI, 122 months to not estimable) in patients who received active surveillance versus 30 months (95% CI, 25–44 months) in those who received systemic therapy. Quality of life at baseline was significantly better in patients who were managed with active surveillance versus systemic therapy [33].

### Consensus recommendation

No consensus was obtained regarding active surveillance in asymptomatic or minimally symptomatic patients. Most of the experts felt the need to start some form of accepted treatment in a metastatic patient and also expressed their concerns about patients who would be lost to follow up, potentially losing out of a chance for systemic treatment and also the cost of radiological tests that is required with such an approach. However, experts recognized need for further studies to determine the optimal selection of patients with mRCC for active surveillance.

### First line systemic therapy: IO + TKI combinations

In an indirect comparison of IO-based combination, the improved PFS and survival advantage of IO combination, particularly for Lenvatinib-Pembrolizumab, are highlighted Table 3.

**Table 3:** First-line IO Combination Trials in mRCC [34–39]

	**CheckMate 214 (Ipi/Nivo)**[34] (***n* = 550 vs n = 546)**	**KEYNOTE-426 (Axi/Pembro)[**35] (***n* = 432 vs *n* = 429)**	**CheckMate 9ER Final analysis (Cabo/Nivo)[**36**] (*n* = 323 vs *n* = 328)**	**CLEAR (Len/Pembro)[**37] **(*n* = 355 vs *n* = 357)**	**JAVELIN Renal 101 (Axi/Ave)[**38**] (*n* = 442 vs *n* = 444)**	**Cabosun (Cabozantinib vs. Sunitinb)**[39] **(*n* = 79 vs. *n* = 78)**
mOS, mo HR (CI)	NR vs 38.4 **0.69** (0.59–0.81)	45.7 vs 40.1 **0.73** (0.60–0.88)	37.7 vs 34.3 **0.70** (0.55–0.90)	NR vs NR **0.66** (0.49–0.88)	NR vs NR **0.80** (0.61–1.02)	26.6 vs. 21.1 0.80 (0.53- 1.21)
Landmark OS 12 mo Landmark OS 24 mo	**83%** vs 78% **71%** vs 61%	**90%** vs 79% **74%** vs 66%	**86%** vs 76% **72%** vs 60% (est)	**90%** vs 79% **79%** vs 70% (est.)	**90%** vs 85%	
mPFS, mo HR (CI)	**12.2** vs 12.3 0.89 (0.76–1.05)	**15.7** vs 11.1 0.68 (0.58–0.80)	**16.6** vs 8.3 0.56 (0.46–0.68)	**23.9** vs 9.2 0.39 (0.32–0.49)	**13.3** vs 8.4 0.69 (0.57–0.82)	8.6 vs. 5.3 0.48 (0.32–0.78)
ORR, %	**39** vs 32	**60** vs 40	**56** vs 28	**71** vs 36	**53** vs 27	**33 vs 12 (Invst)** **20 vs 9 ( Expl)**
CR, %	**11** vs 3	**10** vs 4	**12** vs 5	**16** vs 4	3.8 vs 2	
Med f/u, mo	**55**	**42.8**	**32.9**	**27**	**19**	**21**
Prognostic risk, %						
▪ Favorable	23	32	23	31	21	0
▪ Intermediate	61	55	58	59	61	81
▪ Poor	17	13	19	9	16	19
Prior nephrectomy	82%	83%	69%	74%		72%
Subsequent systemic	Overall (69%)	Overall (69%)	Overall (45%)	Overall (71%)	Overall (82%)	
therapies for sunitinib arm, %	IO (42%)	IO (48%)	IO (34%)	IO (53%)	IO (43%)	
Grade 3 or more Toxicity (%)	46%	76%	65%	82%	71%	68 vs. 65

Immunotherapies are often used in the treatment of aRCC, in addition to anti-angiogenesis therapy. Diarrhoea, hypertension, fatigue, hypothyroidism and reduced appetite were the most common adverse effects reported in the Phase III Keynote-426 trial using Pembrolizumab. In contrast, fatigue, nausea, pruritus, diarrhoea were reported more frequently with Nivolumab in a Phase III study Checkmate 025 trial; and Diarrhoea, hypertension, fatigue, nausea and palmar-plantar erythrodysesthesia syndrome with Avelumab in a Phase III JAVELIN Renal 101 study. In a Phase III CheckMate 214 trial, Ipilimumab reported fatigue, rash, pruritus, nausea and arthralgia [40].

Based on the Pan-Asian ESMO recommendations, patients with advanced disease, regardless of their IMDC prognostic subgroup, should initially be treated with either axitinib and pembrolizumab, Cabozantinib and nivolumab, or Lenvatinib and pembrolizumab [41].

Additionally, in the favourable and intermediate/poor risk categories, NCCN 2022 recommends axitinib + Pembrolizumab, Lenvatinib + Pembrolizumab, and Cabozantinib + Nivolumab, respectively, with nivolumab + Ipilimumab as the recommended treatment in the intermediate/poor risk group [16].

Summary points for IO + TKI combinations:

- ➢ All the IO based combinations had a better OS in comparison to sunitinib
- ➢ IO based combinations showed a significant reduction in the risk of death in the intermediate/poor risk category but no such significant benefit seen in the favourable risk category in comparison to sunitinib
- ➢ Maximum reduction in the risk of death with cabozantinib/nivolumab and lenvatinib/pembrolizumab
- ➢ Greatest PFS benefit (61%) seen with lenvatinib/pembrolizumab

### Consensus recommendations

In the favourable risk category, there was a consensus for the use of axitinib + pembrolizumab and lenvatinib + pembrolizumab as 1^st^ line systemic therapy while there was a near consensus for cabozantinib + nivolumab. Most of the experts highlighted the lack of survival benefit in this subgroup of the combination over the single agent TKIs, and this should be considered while choosing the first line treatment in this subgroup.

In intermediate or poor risk category patients, consensus was obtained for the use of any TKI + IO combinations (Cabozantinib + Nivolumab, Lenvatinib + Pembrolizumab, Axitinib + Pembrolizumab) as well as IO + IO combination (Ipilimumab + Nivolumab) as 1^st^ line systemic therapy, while near consensus was obtained for single agent cabozantinib.

### First line systemic therapy: Single agent TKI

At a median follow-up of 24 months, the phase II CABOSUN study demonstrated a median OS of 26.6 months with cabozantinib compared to 21.2 months with sunitinib (HR = 0.80). When comparing cabozantinib and sunitinib, the median progression-free survival (PFS) was 8.6 months versus 5.3 months, respectively (HR = 0.48, *p* = 0.0008) [38]. Cabozantinib has the highest probability of being the best treatment in terms of PFS (NMA P scores: 0.9481), followed by sunitinib, pazopanib, and tivozanib, according to a Network Meta-Analysis of first-line TKI therapies approved for mRCC [42]. Cabozantinib also significantly increased PFS in intermediate-, and poor-risk categories, according to another network met analysis that indirectly assesses the efficacy of cabozantinib versus standard-of-care (SoC) comparators, prior to the IO era [43].

A meta-analysis comparing sunitinib and pazopanib found that both drugs had comparable PFS (HR = 1.06, *P* = 0.13), OS (HR = 0.92, *P* = 0.29), objective response rate (RR = 1.03, *p* = 0.58), and disease control rate (RR = 1.03, *P* = 0.54). Sunitinib had more cases of severe fatigue, thrombocytopenia, and neutropenia, while pazopanib had more liver toxicity [44].

Though anti-VEGF therapies are generally well tolerated and in use since decades, hypertension, renal insufficiency, Fatigue/asthenia, nausea-vomiting are common side effect seen with TK/VEGF-directed treatment [45]. Aside from VEGF-I, m-Tor inhibitors such as everolimus used in combination with lenvatinib are associated with stomatitis, rash, tiredness, hypercholesterolemia, hypertriglyceridemia, and hyperglycemia [46].

Regarding the favorable risk group, results of recent systematic review suggests a benefit in PFS from IO–TKI compared to sunitinib in this population, but not in OS. Therefore, treatment selection should be made carefully in favorable-risk patients, taking into account other factors (need to define these factors) that may influence treatment decisions.

### Summary points for single agent TKIs

Sunitinib and pazopanib have been shown to have PFS, OS, and ORR that are comparable, while cabozantinib has been shown to have a greater benefit for PFS.

### Pan-Asia ESMO recommendations [41]

Sunitinib, Pazopanib, and are first-line IO alternatives when IO is contraindicated or unavailable. Cabozantinib may also be used to treat IMDC intermediate- and poor-risk disease in patients who are not candidates for first-line IO therapy. Sunitinib or pazopanib are alternatives to IO-based combination therapy in patients with IMDC favourable-risk disease due to the lack of clear superiority of IO-based combinations over sunitinib.

### NCCN 2022 [16]

For single agent TKIs, NCCN recommends, Cabozantinib as a preferred therapy in patients with intermediate/poor risk and as an alternative preferred therapy in patients with favourable risk. It also recommends pazopanib and sunitinib as other preferred therapies for patients in the favourable and intermediate/poor risk categories.

### Consensus recommendations

As a single agent TKI, near consensus was obtained for single agent cabozantinib in patients with intermediate/poor risk category.

### Special circumstances for 1^st^ line treatment

#### When IO is not available/ not affordable or contraindicated

When IO is not available/ not affordable or contraindicated, experts recommended pazopanib/sunitinib as 1^st^ line systemic therapy for favourable risk category and cabozantinib as 1^st^ line systemic therapy for intermediate/poor risk category.

### Disease burden

For disease burden categorization into high or low disease burden, emphasis was laid on the clinical judgement based on the type and nature of symptoms along with risk stratification.

Overall, patients with high disease burden tend to have shorter PFS and OS and hence require rapid disease control to reduce tumour burden and thereby improve the symptoms. Such patients may benefit from TKI/IO combination therapy, due to their superior responses [34–38]. It has also been shown to have a higher ORR than the IO/IO combination, regardless of IMDC criteria [47]. Pembrolizumab/lenvatinib likely has the highest ORR and PFS among the three IO/TKI combinations, as well as the highest rate of grade 3 AEs and discontinuation rate [37].

The high ORRs of lenvatinib plus pembrolizumab (70%) and nivolumab plus cabozantinib (57%) compared to other regimens show that these combinations are particularly well-suited for patients with tumour involvement of organs such as the liver and bones, who typically require a rapid therapeutic response. Patients in good performance status who are able to tolerate the treatment's side effects (such as hypertension and proteinuria) may benefit such combinations to make note is the combination of lenvatinib plus pembrolizumab which has lead to the highest CR rate (16%) documented in the literature [37].

### Consensus recommendations:

- In high disease burden patients, there was consensus towards the use of TKI + IO and near consensus towards the use of pazopanib/sunitinib in the favourable risk group. Almost, similar result was seen in the intermediate/poor risk group where consensus was obtained for TKI + IO combination. However, in this category, near consensus was obtained for IO + IO combination and for cabozantinib but there was no consensus on the use of pazopanib/sunitinib.
- In low disease burden patients, experts recommended use of TKI + IO or pazopanib/sunitinib (consensus) in the favourable risk group while in intermediate/poor risk group patients, TKI + IO, IO + IO combinations or cabozantinib were recommended (consensus).

### Ejection fraction < 45

RCC itself is one of the causative factor for heart failure, in addition to this, small TKIs also have cardiotoxic effect, can lead to hypertension, asymptomatic left ventricular (LV) dysfunction and even congestive heart failure (CHF) have been reported. A meta-analysis published in 2015 found that the relative risk of all grade and high-grade congestive heart failure was significantly higher for patients with TKIs as compared to those without TKIs [47]. In general, to avoid the cardiac side effect of the TKIs, nivolumab plus ipilimumab is considered as the preferred choice of therapy and the same was confirmed by experts.

### Consensus recommendation

In aRCC patients with an ejection fraction of < 45%, there was consensus on the use of IO + IO combinations and near consensus on the use of cabozantinib.

### Second line systemic therapy in accRCC

The choice of second line therapy was based on the best response on the first line therapy and was further stratified based on the time of progression with TKI/IO combinations (Early progression: ≤ 12 months, Late progression: > 12 months) or to single agent TKI (Early progression: ≤ 6 months, Late progression: > 6 months) Table 4.

**Table 4:** Pivotal randomized trials in accRCC post TKI therapy [48–55]

Parameter	RECORD-1 [48, 49]	AXIS [50, 51]	METEOR [52, 53]	CheckMate 025 [54]	LEN EVE [55]
	**Everolimus** **vs** **Placebo**	**Axitinib** **vs** **Sorafenib**	**Cabozantinib** **vs** **Everolimus**	**Nivolumab** **vs** **Everolimus**	**Lenvatinib + Everolimus** **Vs Everolimus**
Patients, n	410	723	658	821	153
MSKCC risk, %					
▪ Good	29	28	46	36	23
▪ Intermediate	56	37	42	49	36
▪ Poor	15	33	13	15	40
Prior TKI	Anti-VEGF	Sunitinib	Anti-VEGF	Anti-VEGF	Anti-VEGF
Line of therapy	2nd or beyond	2nd	2nd or beyond	2nd or 3rd	2nd
ORR, %	2 vs 0	19 vs 9	21 vs 5	25 vs 5	43 vs 3
Median OS, mos	14.8 vs 14.0	20.1 vs 19.2	21.4 vs 17.1	25.0 vs 19.6	25.5 vs 15.4

Summary points: Greater OS and PFS were achieved with lenvatinib and everolimus combination apart from the impressive responses of 35%.

In general, Second-line treatments should take into account the mechanisms of resistance shown with first-line medications and incorporate new approaches to care. Since there are a variety of IO/TKI combinations that can be used in the first-line context for patients with advanced ccRCC, there is a pressing need to standardise the treatment of these patients going forward. In general, for patients who progress on an IO, a TKI or an IO/TKI combination; another anti-VEGFR (Vascular Endothelial Growth Factor Receptor) TKI, or mTOR inhibitor treatment is recommended [56].

### Pan-Asia ESMO recommendations [41]

For second-line treatment, following TKIs, nivolumab or cabozantinib is preferable. Lenvatinib + everolimus is FDA and EMA-approved after TKI failure and could be considered following progression after first-line TKI monotherapy or a TKI in combination with an IO.

In patients already treated with previous two lines of TKI therapy and whose disease has progressed, either nivolumab or cabozantinib can be considered. Sequencing TKI therapy after PD-1-based first-line therapy is related with modest response rates. Therefore, patients should receive a TKI agent that they have not received previously. RCT data to support continued IO inhibition after established progression is sparse, and thus it is not recommended.

### NCCN 2022 recommendation [16]

The NCCN recommends cabozantinib, Nivolumab (both Category 1), and lenvatinib + Everolimus as preferred therapy for subsequent therapy, with Axitinib and Tivozanib as the other recommended category 1 drugs. The NCCN's other recommended regimen includes IO combinations (IO-IO, TKI + IO).

### Consensus recommendations

When axitinib + pembrolizumab was used in the first line, experts came to a consensus that lenvatinib + everolimus or cabozantinib should be the second line treatment options in both early and late progression. Experts also agreed that if more than 12 months have passed since the discontinuation of axitinib + pembrolizumab, re-challenge with a pembrolizumab-based combination could be considered (near consensus).

When lenvatinib plus pembrolizumab was used in the first line, there was consensus on the use of cabozantinib in the second line in both early and late progression. When disease progression occurred after 12 months of stopping lenvatinib + pembrolizumab, a near consensus was reached on the use of a pembrolizumab-based combination.

When disease progression occurred after the use of first-line cabozantinib + nivolumab, experts recommended lenvatinib + everolimus (consensus) in cases of early or late progression. Experts agreed that if disease progression occurs with cabozantinib + nivolumab in first line, re-challenge with any IO-based combination is recommended (consensus) if more than 12 months have passed since the discontinuation of cabozantinib + nivolumab.

In the case of patients who experienced early disease progression while receiving ipilimumab + nivolumab, experts reached a consensus for the use of cabozantinib and a near consensus for lenvatinib + everolimus. If the progression occurred later (after 12 months), there was agreement on using cabozantinib or lenvatinib plus everolimus in the second line. When disease progression occurred, there was near consensus on the use of a nivolumab-based combination after 12 months of discontinuation of first-line ipilimumab + nivolumab.

As shown in Fig. 3, as the patient progressed with cabozantinib in the first line, consensus was reached on the use of nivolumab in the second line, and near consensus was reached on the use of ipilimumab + nivolumab in the second line. If the progression occurred later (> 6 months), there was consensus on the use of nivolumab or lenvatinib + everolimus and near consensus on the use of ipilimumab + nivolumab in the second line.

If sunitinib/pazopanib was used in the first line, in case of early disease progression, a consensus was obtained on nivolumab or ipilimumab + nivolumab in the second line while a near consensus was obtained on cabozantinib or lenvatinib + everolimus combination. When there was a delayed progression (> 6 months) after first line sunitinib/ pazopanib, consensus was obtained on the use of nivolumab in first line while a near consensus was obtained on cabozantinib or ipilimumab + nivolumab or lenvatinib + everolimus.

### Limitation

Few Limitations of Delphi-based consensus include the fact that it is a lengthy procedure that takes at least two rounds of interaction to reach consensus, as well as continual commitment from the experts participating in the consensus who are being asked the same question repeatedly. Genomic mutation is not routinely done in India for the evaluation of prognosis and drug response, hence it was not considered in the consensus development.

## Conclusion

After the availability of multiple novel drugs, sequencing has become much more complex. In some ways, there are no right or wrong answers to sequencing these therapies beyond first-line therapy. The Most important question is if the patients are fit enough to tolerate combination treatment, they should start first-line therapy with a combination immunotherapy with a TKI or another IO and then consider options for sequencing thereafter.

The current article describes comprehensive algorithms for both treatment-naive and pre-treated patients with accRCC. This will assist oncologist in making informed treatment decisions. The algorithmic approach for accRCC management proposed here is dynamic and will need to be revisited as newer therapeutic agents become available in our country.

### Supplementary Information

**Additional file 1.**

## Data Availability

The datasets used and/or analysed during the current study available from the corresponding author on reasonable request.

## Abbreviations

aRCC: Advanced Renal Cell Carcinoma
TKIs: Tyrosine kinase inhibitors
IOs: Immune-oncologicals
ccRCC: Clear cell RCC
VEGF: Vascular endothelial growth factor
IFN- α: Interferon- α
mTOR: Mammalian target of rapamycin
ICIs: Immune check point inhibitors
IMDC: International Metastatic renal cell carcinoma Database Consortium
MSKCC: Memorial Sloan- Kettering Cancer Center
LDH: Lactate dehydrogenase
PFS: Progression-free survival
OS: Overall Survival
