# Supplementary material for: ExPert ConsEnsus on the management of Advanced clear-cell RenaL celL carcinoma: INDIAn Perspective (PEARL-INDIA)
Source: BMC Cancer. 2023 Aug 9;23:737. doi: 10.1186/s12885-023-11237-y (PMC10413514; doi:10.1186/s12885-023-11237-y)
Supplement: Supplementary file 1 — Additional file 1. [file 12885_2023_11237_MOESM1_ESM.docx]

**Supplementary appendix I**

**Class of recommendation and level of evidence for all clinical statements**

**Consensus:** Statements achieving a mean score of **7.00 or higher** with no more than 1 outlier

**Near consensus:** Statements achieving a mean score of **6.50 or higher** with no more than 2 outliers

**No consensus:** Statements NOT meeting the criteria of consensus or near consensus

Outlier defined as any rating ≥2 Likert points from the mean in either direction

|  | **Question** | **Average score** | **Outlier** | **Consensus** | **COR and LOE** |
| --- | --- | --- | --- | --- | --- |
| **Risk stratification** | | | | | |
| 1 | The International Metastatic RCC Database Consortium (IMDC) criteria is more clinically relevant for risk stratification | 8.54 | 0 | Consensus | I-A |
| 2 | The Memorial Sloan-Kettering Cancer Center (MSKCC) criteria is more clinically relevant for risk stratification | 5.38 | 2 | No consensus | III-A |
| **First line therapy** | | | | | |
|  | **Favourable risk category** | | | |  |
| 1 | Active surveillance can be offered to treatment-naïve patients who are asymptomatic or minimally symptomatic | 6 | 2 | No consensus | III-B |
| 2 | Axitinib + pembrolizumab is the preferred therapy in clinical practice in symptomatic patients | 7.54 | 1 | Consensus | I-A |
| 3 | Lenvatinib + pembrolizumab is the preferred therapy in clinical practice in symptomatic patients | 7.54 | 0 | Consensus | I-A |
| 4 | Cabozantinib + nivolumab is the preferred therapy in clinical practice in symptomatic patients | 6.77 | 1 | Near consensus | II-A |
| 5 | Ipilimumab + nivolumab is the preferred therapy in clinical practice in symptomatic patients | 4.92 | 4 | No consensus | III-B |
| 6 | Cabozantinib is an option to IO based combinations when immune therapy is not available or is contraindicated | 5.69 | 5 | No consensus | III-C |
| 7 | Single agent TKI (Pazopanib/sunitinib) is the preferred therapy in clinical practice in symptomatic patients irrespective of the affordability and availability | 5.46 | 5 | No consensus | III-A |
|  | **Intermediate/poor risk category** | | | |  |
| 1 | Cabozantinib + nivolumab is the preferred therapy in clinical practice in symptomatic patients | 8.08 | 1 | Consensus | I-A |
| 2 | Lenvatinib + pemrolizumab is the preferred therapy in clinical practice in symptomatic patients | 8.15 | 0 | Consensus | I-A |
| 3 | Axitinib + pemrolizumab is the preferred therapy in clinical practice in symptomatic patients | 7.85 | 1 | Consensus | I-A |
| 4 | Ipilimumab + nivolumab is the preferred therapy in clinical practice in symptomatic patients | 7.23 | 1 | Consensus | I-A |
| 5 | Axitinib + avelumab is the preferred therapy in clinical practice in symptomatic patients | 6 | 0 | No consensus | III-A |
| 6 | Cabozantinib is the preferred therapy in clinical practice in symptomatic patients | 6.69 | 1 | Near consensus | II-A |
| 7 | Single agent TKI (Pazopanib/sunitinib) is the preferred therapy in clinical practice in symptomatic patients irrespective of the affordability and availability | 3.00 | 1 | No consensus | III-A |
|  | **First line therapy in special circumstances** | | | |  |
| **Preferred clinical practice therapy when immune therapy is not available/not affordable or is contraindicated** | | | | | |
|  | ***Favourable risk category*** |  |  |  |  |
| 1 | Pazopanib/ Suntinib is the preferred therapy | 8.31 | 1 | Consensus | I-A |
|  | ***Intermediate/poor risk category*** |  |  |  |  |
| 1 | Pazopanib/ Suntinib is the preferred therapy | 5.77 | 4 | No consensus | III-A |
| 2 | Cabozantinib is the preferred therapy | 7.31 | 1 | Consensus | I-A |
| **Preferred class of therapy/therapy in clinical practice in high disease burden: Symptomatic and/or rapidly progressive disease requiring rapid control** (In disease burden categorization stated above, type and nature of symptoms to be judged clinically along with risk stratification while managing the patient) | | | | | |
|  | ***Favourable risk category*** |  |  |  |  |
| 1 | TKI (Pazopanib/sunitinib) is the preferred therapy | 7.08 | 2 | Near consensus | II-C |
| 2 | IO+TKI is the preferred class of therapy | 8 | 0 | Consensus | I-C |
| 3 | IO+IO is the preferred class of therapy | 5.31 | 5 | No consensus | III-C |
|  | ***Intermediate/poor risk category*** |  |  |  |  |
| 1 | TKI (Pazopanib/sunitinib) is the preferred therapy | 5.38 | 8 | No consensus | III-C |
| 2 | TKI (Cabozantinib) is the preferred therapy | 7.46 | 2 | Near consensus | II-C |
| 3 | IO+TKI is the preferred therapy | 8.46 | 0 | Consensus | I-C |
| 4 | IO+IO is the preferred class of therapy | 6.85 | 1 | Near consensus | II-C |
| **Preferred therapy/class of therapy in clinical practice in low disease burden** | | | | | |
|  | ***Favourable risk category*** |  |  |  |  |
| 1 | TKI (Pazopanib/sunitinib) is the preferred therapy | 8.31 | 0 | Consensus | I-C |
| 2 | IO+TKI is the preferred class of therapy | 7.77 | 1 | Consensus | I-C |
| 3 | IO+IO is the preferred class of therapy | 5.08 | 5 | No consensus | III-C |
|  | ***Intermediate/poor risk category*** |  |  |  |  |
| 1 | TKI (Pazopanib/sunitinib) is the preferred therapy | 5.54 | 6 | No consensus | III-C |
| 2 | TKI (Cabozantinib) is the preferred therapy | 7.38 | 1 | Consensus | I-C |
| 3 | IO+TKI is the preferred class of therapy | 8 | 1 | Consensus | I-C |
| 4 | IO+IO is the preferred class of therapy | 7.08 | 1 | Consensus | I-C |
| **Preferred clinical practice therapy in cardiovascular comorbidity (ejection fraction <45 %) irrespective of risk stratification** | | | | | |
| 1 | IO+TKI is preferred | 6.31 | 1 | No consensus | III-C |
| 2 | IO+IO is preferred | 7.15 | 1 | Consensus | I-C |
| 3 | Single agent TKI (Cabozantinib) is preferred | 6.62 | 1 | Near consensus | II-C |
| 4 | Single agent TKI (Pazopanib) is preferred | 6.23 | 2 | No consensus | III-C |
| 5 | Single agent TKI (Sunitinib) is preferred | 4.54 | 1 | No consensus | III-C |

| **Second line therapy** | | | | |  |
| --- | --- | --- | --- | --- | --- |
| **Patients who have taken axitinib + pembrolizumab in first line** | | | | | |
|  | **Question** | **Average score** | **Outlier** | **Consensus** | **COR and LOE** |
| 1 | Cabozantinib is the preferred therapy in clinical practice in cases of early progression (≤ 12 months) | 7.62 | 1 | Consensus | I-A |
| 2 | Cabozantinib is the preferred therapy in clinical practice in cases of late progression (> 12 months) | 7.77 | 0 | Consensus | I-A |
| 3 | Nivolumab is the preferred therapy in clinical practice in cases of early progression (≤ 12 months) | 3.69 | 4 | No consensus | III-C |
| 4 | Nivolumab is the preferred therapy in clinical practice in cases of choice in late progression (> 12 months) | 4.69 | 3 | No consensus | III-C |
| 5 | Lenvatinib + Everolimus is the preferred therapy in clinical practice in cases of in early progression (≤ 12 months) | 7.46 | 1 | Consensus | I-C |
| 6 | Lenvatinib + Everolimus is the preferred therapy in clinical practice in cases of late progression (> 12 months) | 7.46 | 1 | Consensus | I-C |
| 7 | Ipilimumab+ nivolumab is the preferred therapy in clinical practice in cases of early progression (≤ 12 months) | 4.38 | 4 | No consensus | III-C |
| 8 | Ipilimumab+ nivolumab is the preferred therapy in clinical practice in cases of late progression (> 12 months) | 4.92 | 1 | No consensus | III-C |
| 9 | Re-challenge with a pemrolizumab based combination (IO+TKI) is preferred in clinical practice (after > 12 months of discontinuation) when the patient has obtained a reasonable clinical response with the first therapy | 6.92 | 1 | Near consensus | II-C |
|  | **Patients who have taken lenvatinib + pemrolizumab in first line** | | | |  |
| 1 | Cabozantinib is the preferred therapy in clinical practice in cases of early progression (≤ 12 months) | 7.85 | 1 | Consensus | I-C |
| 2 | Cabozantinib is the preferred therapy in clinical practice in cases of late progression (> 12 months) | 7.31 | 1 | Consensus | I-C |
| 3 | Nivolumab is the preferred therapy in clinical practice in cases of early progression (≤ 12 months) | 3.38 | 1 | No consensus | III-C |
| 4 | Nivolumab is the preferred therapy in clinical practice in cases of late progression (> 12 months) | 4.62 | 3 | No consensus | III-C |
| 5 | Axitinib is the preferred therapy in clinical practice in cases of late progression (> 12 months) | 5.15 | 1 | No consensus | III-C |
| 6 | Ipilimumab+ nivolumab is the preferred therapy in clinical practice in cases of early progression (≤ 12 months) | 4.00 | 3 | No consensus | III-C |
| 7 | Ipilimumab+ nivolumab is the preferred therapy in clinical practice in cases of in late progression (> 12 months) | 5.08 | 3 | No consensus | III-C |
| 8 | Re-challenge with a pemrolizumab based combination (IO+TKI) is preferred in clinical practice (after > 12 months of discontinuation) when the patient has obtained a reasonable clinical response with the first therapy | 6.69 | 1 | Near consensus | II-C |
|  | **Patients who have taken cabozantinib + nivolumab in first line** | | | |  |
| 1 | Lenvatinib + Everolimus is the preferred therapy in clinical practice in cases of early progression (≤ 12 months) | 7.54 | 1 | Consensus | I-C |
| 2 | Lenvatinib + Everolimus is the preferred therapy in clinical practice in cases of late progression (> 12 months) | 7.85 | 0 | Consensus | I-C |
| 3 | Axitinib is the preferred therapy in clinical practice in cases of early progression (≤12 months) | 4.92 | 1 | No consensus | III-C |
| 4 | Axitinib is the preferred therapy in clinical practice in cases of late progression (> 12 months) | 5.77 | 0 | No consensus | III-C |
| 5 | Re-challenge with an IO based combination is preferred in clinical practice (after >12 months of discontinuation when the patient had a reasonable clinical response with the first therapy and the drug was electively stopped with no major toxicity concerns | 7.46 | 1 | Consensus | I-C |
| 6 | Re-challenge with single agent nivolumab is preferred in clinical practice (after > 12 months of discontinuation) when the patient has obtained a reasonable clinical response with the first therapy | 5.69 | 3 | No consensus | III-C |
|  | **Patients who have taken ipilimumab+ nivolumab in first line** | | | |  |
| 1 | Cabozantinib is the preferred therapy in clinical practice in cases of early progression (≤ 12 months) | 7.46 | 0 | Consensus | I-C |
| 2 | Cabozantinib is the preferred therapy in clinical practice in cases of late progression (> 12 months) | 7.31 | 0 | Consensus | I-C |
| 3 | Lenvatinib + Everolimus is the preferred therapy in clinical practice in cases of early progression (≤ 12 months) | 6.92 | 1 | Near consensus | II-C |
| 4 | Lenvatinib + Everolimus is the preferred therapy in clinical practice in cases of late progression (> 12 months) | 7.38 | 0 | Consensus | I-C |
| 5 | Axitinib is the preferred therapy in clinical practice in cases of early progression (≤ 12 months) | 5.23 | 0 | No consensus | III-C |
| 6 | Axitinib is the preferred therapy in clinical practice in cases of late progression (> 12 months) | 5.85 | 1 | No consensus | III-C |
| 7 | Re-challenge with a nivolumab based combination (IO+IO or IO+TKI) is preferred in clinical practice (after > 12 months of discontinuation) when the patient has obtained a reasonable clinical response with the first therapy | 6.54 | 1 | Near consensus | II-C |
| 8 | Re-challenge with single agent nivolumab is preferred in clinical practice (after > 12 months of discontinuation) when the patient has obtained a reasonable clinical response with the first therapy | 5.69 | 3 | No consensus | III-C |
|  | **Patients who have taken cabozantinib in first line** |  |  |  |  |
| 1 | Nivolumab is the preferred therapy in clinical practice in cases of early progression (≤ 6 months) | 7.54 | 1 | Consensus | I-C |
| 2 | Nivolumab is the preferred therapy in clinical practice in cases of late progression (> 6 months) | 7.62 | 1 | Consensus | I-C |
| 3 | Lenvatinib + Everolimus is the preferred therapy in clinical practice in cases of early progression (≤ 6 months) | 5.54 | 5 | No consensus | III-C |
| 4 | Lenvatinib + Everolimus is the preferred therapy in clinical practice in cases of late progression (> 6 months) | 7.08 | 0 | Consensus | I-C |
| 5 | Axitinib is the preferred therapy in clinical practice in cases of early progression (≤ 6 months) | 4.08 | 0 | No consensus | III-C |
| 6 | Axitinib is the preferred therapy in clinical practice in cases of late progression (> 6 months) | 5.15 | 1 | No consensus | III-C |
| 7 | Ipilimumab+ nivolumab is the preferred therapy in clinical practice in cases of early progression (≤ 6 months) | 6.77 | 1 | Near consensus | II-C |
| 8 | Ipilimumab+ nivolumab is the preferred therapy in clinical practice in cases of late progression (> 6 months) | 6.92 | 1 | Near consensus | II-C |
|  | **Patients who have taken sunitinib/pazopanib in first line** | | | |  |
| 1 | Cabozantinib is the preferred therapy in clinical practice in cases of early progression (≤ 6 months) | 6.54 | 0 | Near consensus | II-C |
| 2 | Cabozantinib is the preferred therapy in clinical practice in cases of late progression (> 6 months) | 6.92 | 0 | Near consensus | II-C |
| 3 | Nivolumab is the preferred therapy in clinical practice in cases of early progression (≤ 6 months) | 7.31 | 0 | Consensus | I-C |
| 4 | Nivolumab is the preferred therapy in clinical practice in cases of late progression (> 6 months) | 7.62 | 0 | Consensus | I-C |
| 5 | Lenvatinib + Everolimus is the preferred therapy in clinical practice in cases of early progression (≤ 6 months) | 6.69 | 1 | Near consensus | II-C |
| 6 | Lenvatinib + Everolimus is the preferred therapy in clinical practice in cases of late progression (> 6 months) | 6.85 | 1 | Near consensus | II-C |
| 7 | Axitinib is the preferred therapy in clinical practice in cases of early progression (≤ 6 months) | 4 | 2 | No consensus | III-C |
| 8 | Axitinib is the preferred therapy in clinical practice in cases of late progression (> 6 months) | 5.77 | 3 | No consensus | III-C |
| 9 | Axitinib is an option in clinical practice in cases of early progression (≤ 6months) | 5.62 | 4 | No consensus | III-C |
| 10 | Axitinib is an option in clinical practice in cases of late progression > 6 months) | 7.23 | 0 | Consensus | I-C |
| 11 | Ipilimumab+ nivolumab is the preferred therapy in clinical practice in cases of early progression (≤ 6 months) | 7.08 | 1 | Consensus | I-C |
| 12 | Ipilimumab+ nivolumab is the preferred therapy in clinical practice in cases of late progression (> 6 months) | 6.92 | 1 | Near consensus | II-C |
